# Supplementary material for: Sepsis research in Canada: An environmental scan of sepsis investigators, research, and funding
Source: PLOS Glob Public Health. 2025 Apr 29;5(4):e0003606. doi: 10.1371/journal.pgph.0003606 (PMC12040209; doi:10.1371/journal.pgph.0003606)
Supplement: S1 Text — (DOCX) [file pgph.0003606.s001.docx]

# Supporting Information

**S1 Text. Full Questionnaire.**

1. Your Name [open-ended]:
2. Your Email [open-ended]:
3. Which gender do you identify with? (Select one option)
   1. Man
   2. Woman
   3. Non-binary
   4. Prefer not to answer
4. Please select the option(s) that best describe your racial background (Select all that apply) [Caption: Race is a construct that is part of our social identity, which impacts the perspectives and experiences that guide our contributions to research. Ensuring diversity and eliminating disparities is important for producing equitable and representative research. Source: <https://www.cihi.ca/sites/default/files/document/guidance-and-standards-for-race-based-and-indigenous-identity-data-en.pdf>]
   1. Black/Caribbean/African
   2. East Asian (e.g. Chinese, Japanese, Korean)
   3. South Asian (e.g. Pakistani, Indian, Bangladeshi)
   4. Southeast Asian (e.g. Filipino, Indonesian, Vietnamese)
   5. Hispanic/Latin American
   6. Middle Eastern/West Asian (e.g. Arab, Persian, Afghan, Kurdish, Turkish)
   7. White/European
   8. Prefer not to answer
   9. Other [open-ended]
5. Do you identify as First Nations, Inuk/Inuit, and/or Metis? (Select all that apply)
   1. Yes, First Nations
   2. Yes, Inuk/Inuit
   3. Yes, Metis
   4. No
   5. Do not know
   6. Prefer not to answer
6. Which province/territory are you located in?
   1. Alberta
   2. British Columbia
   3. Manitoba
   4. New Brunswick
   5. Newfoundland and Labrador
   6. Northwest Territories
   7. Nova Scotia
   8. Nunavut
   9. Ontario
   10. Prince Edward Island
   11. Quebec
   12. Saskatchewan
   13. Yukon
7. What is your academic position? Please select from the following [dropdown]:
   1. Professor Emeritus
   2. Professor
   3. Associate Professor
   4. Assistant Professor
   5. Clinical Professor
   6. Clinical Associate Professor
   7. Clinical Assistant Professor
   8. Clinical Scholar/Lecturer
8. Where is your primary academic appointment/institution? [open-ended]
9. Are you a member of the Sepsis Canada Network? <https://www.sepsiscanada.ca/>
   1. Yes [skips to question 11]
   2. No [proceeds to question 10]
10. If not, are you interested in becoming a member?
    1. Yes
    2. No
11. How many years have you been involved in sepsis and/or sepsis-related research? [dropdown]
    1. 0-4
    2. 5-10
    3. 11-20
    4. 20+
12. How many sepsis-related publications (full journal articles, book chapters but not abstracts) do you have in the past 10 years? [dropdown]
    1. 0-4
    2. 5-10
    3. 11-25
    4. 25+
13. Are you currently involved in sepsis research?
    1. Yes [proceeds to question 14]
    2. No [end of survey]
14. How many people are currently on your research team (includes technicians, research coordinators)? [dropdown]
    1. 1-5
    2. 6-10
    3. 11-20
    4. 20+
15. How many post-docs & graduate student trainees are part of your research team? [dropdown]
    1. 0
    2. 1-4
    3. 5-10
    4. 10+
16. How many undergraduate trainees are part of your research team? [dropdown]
    1. 0
    2. 1-2
    3. 3-5
    4. 5+
17. How many patient partners are part of your research team? [dropdown]
    1. 0
    2. 1-2
    3. 3-5
    4. 5+
18. What is your primary research focus? Please select all that apply:
    1. Sepsis prevention
    2. Sepsis identification
    3. Sepsis treatment/management
    4. Post-sepsis effects/Post-sepsis syndrome
    5. Sepsis recovery
    6. Sepsis epidemiology
    7. Sepsis pathophysiology
    8. Sepsis education
    9. Sepsis quality improvement
    10. Preclinical (animal) sepsis model
    11. Sepsis-related health policy
    12. Other [open-ended]
19. Does your research consider any of the following groups? Please select all that apply:
    1. Neonates (birth – first hospital discharge)
    2. Infants (0-1 year)
    3. Children (1-18 years)
    4. Over 65 years of age
    5. Biological sex effects
    6. Gender-related sepsis research
    7. Social determinants of health
    8. Equity, Diversity, Decolonization, and Inclusion
20. Briefly describe your current sepsis-related research projects [open-ended]:
