# Supplementary material for: Sepsis research in Canada: An environmental scan of sepsis investigators, research, and funding
Source: PLOS Glob Public Health. 2025 Apr 29;5(4):e0003606. doi: 10.1371/journal.pgph.0003606 (PMC12040209; doi:10.1371/journal.pgph.0003606)
Supplement: S1 Data — (DOCX) [file pgph.0003606.s002.docx]

# Supporting Information

**S1 Data. Thematic Analysis of Research Focus: CIHR Pillars.**

Biomedical

The data revealed a diverse array of themes within the biomedical research, focused on understanding sepsis pathophysiology, exploring novel interventions, and uncovering diagnostic and prognostic markers.

1. Pharmacokinetics and Therapeutics: Investigations into antimicrobial pharmacokinetics and dosing in septic shock, as well as the potential benefits of endocannabinoid modulation.
2. Immunology and Microbiology: Studies on immunology and microbiology/microbiome of sepsis pathogenesis offer insights into the intricate interactions between microbial agents and host responses.
3. Novel Therapies and Mechanisms: Exploration of the apelinergic system’s potential in preclinical sepsis models and the study of novel analogs of endogenous ligands like Apelin and Elabela. Additional work on understanding of sepsis’s molecular mechanisms including the VWF/ADAMTS13 axis.
4. Pathophysiology and Consequences: Investigations into acute lung injury, mechanisms underlying acute kidney injury, and sepsis-induced myocardial dysfunction were studied to the understand organ-specific impacts of sepsis.
5. Diagnostic Markers and Biomarkers: Research efforts into diagnostic aspects, including the pursuit of sepsis endotypes and early diagnosis markers. Utility of blood cultures, predictors of bacteremia, genetics, biomarkers, and gender-related differences underscores the quest for better diagnostic accuracy and personalized treatment.
6. Interdisciplinary Exploration: The intersection of machine learning, multi-omics integration, and molecular approaches in identifying diagnostic markers for bacterial sepsis reflects interdisciplinary collaboration to advance diagnostic capabilities.

Clinical

Various themes emerged under the clinical research theme including interventional studies and clinical trials, all aimed at improving patient outcomes:

1. Interventions and Therapies: Clinical research efforts encompass a multitude of interventions targeting sepsis-associated complications. These include improving acute kidney injury outcomes in adult sepsis, conducting sepsis-related clinical trials (RCTs) to assess therapies, and evaluating the impact of therapeutic approaches for pneumonia treatment and prevention. The potential benefits of Vitamin C for sepsis and COVID-19, highlight efforts to optimize treatment strategies for critically ill patients.
2. Pathophysiology and Mechanisms: Respondents examined factors such as age, biological sex, and exercise impact on inflammation resolution and endothelial barrier function. The pursuit of a better understanding of pathophysiological aspects also extended to the exploration of diagnostic and prognostic aspects of COVID-19.
3. Specialized Patient Populations: There were a number of efforts to study sepsis in neonates and infants. Efforts to identify optimal treatments for neonatal sepsis, as well as assessing newborn sepsis prediction, illustrated the unique challenges in pediatric care.
4. Clinical Trials and Precision Medicine: A strong emphasis on clinical trials and precision medicine was evident, with studies like PRIMED focusing on precision medicine for pediatric appendicitis and sepsis sub-groups. Initiatives such as PRoMPT BOLUS and SQUEEZE Trial highlighted the drive to optimize treatment strategies using early goal-directed approaches and fluid sparing strategies.
5. Monitoring and Biomarkers: Hemodynamic monitoring, sepsis trigger tools, and the use of biomarkers to personalize antibiotic treatment duration reflect the importance of accurate monitoring and diagnostic tools to guide clinical decisions.
6. Organ-Specific Impacts: The impact of sepsis on specific organs was explored, with studies investigating sepsis-induced encephalopathy, optimal dosing of antibiotics in sepsis and ECMO, and the role of MSC therapy in addressing septic shock and metabolomics mechanisms.
7. Infection Control and Stewardship: The utilization of antimicrobials and stewardship efforts were highlighted, indicating a commitment to optimizing infection control strategies and preserving the effectiveness of antibiotics.

Health Services

The data highlights a range of themes within health services research aimed at improving sepsis management, awareness, and patient outcomes:

1. Educational Initiatives: Efforts are being made to enhance sepsis awareness and knowledge through various educational approaches. These include scoping reviews, experienced-based co-design studies, and the development of educational modules. The establishment of platforms like the "Lifting Sepsis" training platform and initiatives to train outcome assessors reflect a sepsis-related education and awareness.
2. Advocacy and Awareness: Many researchers are actively engaged in advocating for sepsis research and management. This is evident in activities such as awareness-raising, policy promotion, and development of effective communication strategies. The focus on sepsis guidelines and knowledge translation (KT) initiatives underscored the importance of disseminating evidence-based practices to healthcare providers and the public.
3. Patient Engagement and Recovery: Patient-centered research is evident through studies exploring the impacts of engaging with patient advisory councils on long-term recovery for sepsis survivors and their families. Quality improvement initiatives, especially in low- and middle-income countries (LMICs), highlight the strategies to enhance post-discharge care and reduce mortality rates.
4. Guidelines and Resource Utilization: The creation of guidelines specific to pediatric sepsis and the exploration of how sepsis affects future healthcare resource utilization underscore standardization in care and optimizing resource allocation.
5. Barriers and Strategies: Researchers are conducting in-depth investigations into barriers to optimal sepsis care. This includes literature searches, surveys, and key informant interviews to identify challenges and strategies for mitigating them.
6. Long-Term Effects: Studies examining the impacts of COVID-19 as a cause of severe sepsis and investigating post-sepsis syndrome (Long COVID) illustrated a commitment to understanding the broader health implications of sepsis.

Population Health

The data reveals several key themes within the realm of population health research pertaining to sepsis, highlighting efforts to understand the broader societal and contextual factors influencing sepsis outcomes:

1. Patient-Centered Approaches: Population health research in sepsis incorporates patient-centric perspectives, as evidenced by the study of engaging with patient advisory councils. By investigating the long-term recovery experiences of sepsis survivors and their families, researchers aim to gain insights into the holistic impact of sepsis beyond immediate medical care.
2. Global Health Approach to Neonatal Sepsis: The data highlights a global health perspective in studying neonatal sepsis, encompassing both immunology and epidemiology. This theme signifies a commitment to addressing neonatal sepsis within a broader international context, recognizing diverse healthcare systems and populations.
3. Epidemiology and Outcomes: The focus on epidemiology, creating metadata catalogs for sepsis research, and identifying outcome differences across regions underscores the significance of understanding the distribution and impact of sepsis across diverse populations.
4. Regional Outcome Variation Study: A project aiming to identify outcome differences across provinces further reinforces the focus on regional disparities.
