# Supplementary material for: Sepsis research in Canada: An environmental scan of sepsis investigators, research, and funding
Source: PLOS Glob Public Health. 2025 Apr 29;5(4):e0003606. doi: 10.1371/journal.pgph.0003606 (PMC12040209; doi:10.1371/journal.pgph.0003606)
Supplement: Attachment — Submitted filename: PLOS Global Public Health_Response to Reviewers.docx [file pgph.0003606.s004.docx]

**PLOS Global Public Health – Authors’ Response**

Editor Comments:

1. Please provide additional details regarding participant consent. In the ethics statement in the Methods and online submission information, please ensure that you have specified (1) whether consent was informed and (2) what type you obtained (for instance, written or verbal, and if verbal, how it was documented and witnessed). If your study included minors, state whether you obtained consent from parents or guardians. If the need for consent was waived by the ethics committee, please include this information. If you are reporting a retrospective study of medical records or archived samples, please ensure that you have discussed whether all data were fully anonymized before you accessed them and/or whether the IRB or ethics committee waived the requirement for informed consent. If patients provided informed written consent to have data from their medical records used in research, please include this information.

Thank you for the suggestion. Participants were provided details on the study purpose and aims, and the fact that participation was voluntary, in the recruitment email sent to all participants. Consent was implied upon survey completion and Hamilton Integrated Research Ethics Board waived the need for formal consent. We have updated the Methods section with this information.

2.  Please provide an Author Summary. This should appear in your manuscript between the Abstract (if applicable) and the Introduction, and should be 150–200 words long. The aim should be to make your findings accessible to a wide audience that includes both scientists and non-scientists. Sample summaries can be found on our website under Submission Guidelines:
https://journals.plos.org/globalpublichealth/s/submission-guidelines#loc-parts-of-a-submission.

We have added an Author Summary to the manuscript.

3. Figure 2: please (a) provide a direct link to the base layer of the map (i.e., the country or region border shape) and ensure this is also included in the figure legend; and (b) provide a link to the terms of use / license information for the base layer image or shapefile. We cannot publish proprietary or copyrighted maps (e.g. Google Maps, Mapquest) and the terms of use for your map base layer must be compatible with our CC-BY 4.0 license.

Note: if you created the map in a software program like R or ArcGIS, please locate and indicate the source of the basemap shapefile onto which data has been plotted.

If your map was obtained from a copyrighted source please amend the figure so that the base map used is from an openly available source. Alternatively, please provide explicit written permission from the copyright holder granting you the right to publish the material under our CC-BY 4.0 license.

Please note that the following CC BY licenses are compatible with PLOS license: CC BY 4.0, CC BY 2.0 and CC BY 3.0, meanwhile such licenses as CC BY-ND 3.0 and others are not compatible due to additional restrictions.

If you are unsure whether you can use a map or not, please do reach out and we will be able to help you. The following websites are good examples of where you can source open access or public domain maps: 
* U.S. Geological Survey (USGS) - All maps are in the public domain. (http://www.usgs.gov) 
* PlaniGlobe - All maps are published under a Creative Commons license so please cite “PlaniGlobe, http://www.planiglobe.com, CC BY 2.0” in the image credit after the caption. (http://www.planiglobe.com/?lang=enl) 
* Natural Earth - All maps are public domain. (http://www.naturalearthdata.com/about/terms-of-use/)

We have changed the base layer of the map to be sourced from GADM (<https://gadm.org/maps/CAN.html>) and updated Figure 2 and its caption accordingly. According to the GADM licence information: “The data are freely available for academic use and other non-commercial use. Redistribution or commercial use is not allowed without prior permission. Using the data to create maps for publishing of academic research articles is allowed. Thus you can use the maps you made with GADM data for figures in articles published by PLoS, Springer Nature, Elsevier, MDPI, etc. You are allowed (but not required) to publish these articles (and the maps they contain) under an open license such as CC-BY as is the case with PLoS journals and may be the case with other open access articles” (<https://gadm.org/license.html>). Please let us know if this would align with the journal’s requirements.

4. Please review your reference list to ensure that it is complete and correct. If you have cited papers that have been retracted, please include the rationale for doing so in the manuscript text, or remove these references and replace them with relevant current references. Any changes to the reference list should be mentioned in the rebuttal letter that accompanies your revised manuscript. If you need to cite a retracted article, indicate the article’s retracted status in the References list and also include a citation and full reference for the retraction notice.

We have noted any changes to the reference list in our response to the reviewer’s comments below.

Reviewer Comments:

INTRODUCTION:

Line 44 and line 46: indicate that these are annual (?) estimates for case and death counts and healthcare costs.

Response:
Yes, both these estimates are based on annual rates. However, the annual deaths were based on Stats Canada data collected between 2007 and 2011, which the healthcare costs estimates were based on Ontario data gathered between April 1, 2012, and March 31, 2016. The sentence has been revised to clearly reflect that these were annual estimates.

Line 48-51, up to and including "....2017.": The Canada specific information seems out of place in this paragraph. I would move this info into the subsequent paragraph where you talk about why Sepsis Canada was formed. The line about CMAJ can probably be deleted entirely.
Last paragraph of the Introduction should be edited to include that one aim was assessing extent of investment in sepsis research in Canada.

Response:
We have revised the paragraph to remove the statement on Canada, and the CMAJ reference. And we also included the statement in the last paragraph assessing extent of investment in sepsis research in Canada.

METHODS

Is there a chance that there are eligible participants identified via dissemination through twitter, the CCCTG, and the CCCTBG, who would NOT have been captured via the PubMed search that was done to look for participants? If so, this needs to be clarified under Participants.

Response:
We appreciate the reviewer’s insightful question regarding the potential for participants to be identified through dissemination via Twitter, the CCCTG, and the CCCTBG, who may not have been captured through the PubMed search. Unfortunately, due to the anonymous nature of our survey, we were unable to determine whether responses originated from our email solicitation or social media dissemination. In both cases, the same survey link was provided, and no identifying information was collected to maintain participant anonymity. However, given the broad dissemination strategy employed, it is possible that some participants engaged with the survey through social media or professional networks rather than through direct email contact. We have clarified this point in the Survey Administration section to ensure transparency.

How did you go about determining who were faculty vs. not in the author lists for articles found by your PubMed search? This seems like a lot of work.

Response:
Thank you for recognizing the effort involved in this process. Identified individuals were cross-referenced using their institutional websites (e.g., their university’s website) to determine their faculty status.

Under "Survey Development" - Briefly describe details around i) demographics, and ii) sepsis research (beyond pubs, research team, years active).

Response:
Thank you for your suggestion. Details on demographics and sepsis research (beyond publications, research team, and years active) are provided in the Full Questionnaire, which can be found in the appendix.

Provide the link to the CIHR funding database if it is publicly available.

Response:
Thank you. A reference has now been provided in the Methods section.


RESULTS

The Methods indicate that eligible participants were in part identified via a PubMed search. How many articles did you get with your search to be able to identify the 155 people you sent the survey to to?

Response:
As noted in the Methods, “we conducted a PubMed search using the keyword terms ‘sepsis’ and ‘Canada’ with a date range of 2003–2023.” This search yielded 4000+ articles, from which we simultaneously extracted the author list and cross-referenced the authors, continuing until no new authors emerged. We selected only those with a faculty position at a Canadian institution. We have added this clarification to the Methods section.

Do you have a sense of gender/academic positions of these eligible folks, and how those who didn't respond differ in terms of these characteristics compared to those who did respond?

Response:
Thank you for your thoughtful question. We did not want to make any assumptions about gender or academic positions of eligible participants who did not respond. Therefore, we cannot determine how non-respondents may differ from respondents. Our analysis is based solely on the information provided by those who completed the survey.

Did all participants answer all questions? Or were there blanks?

Response:
Thank you for your question. As mentioned in the Results, “After removing 4 duplicates/blanks, we had a total of 69 valid responses.” There were no partial responses.

Line 183/184: include numerators and denominators, not just percentages.

Response:
The numerator and denominators are included as fractions.

Table 1: footnote indicates that one participants had a cross appointment in Portugal - risks identifying participant, could just say "abroad" as opposed to Portugal.

Response:
That's a great point. We have removed the reference to Portugal.

Line 240-247 incl Table 2: How many participants fall into each CIHR pillar?

Response:
Thank you for your question. We have provided more granular data in Figure 4, where we identify the specific research topics and the number of respondents studying each topic. In Table 2, the data was extracted based on written responses, and researchers could be studying topics that span multiple CIHR themes. As a result, assigning strict numerical values to each pillar would not add meaningful value.

Table 1: Add a column for "Total" across all provinces.

Response:
We appreciate the suggestion. We have added a "Total" column across all provinces in Table 1.

Figure 3: This figure seems unnecessary - all this info is in Table 1. Would remove.

Response:

We have removed Figure 3 and its caption, and renumbered subsequent figures and figure captions accordingly.

Figure 4: I would order this by frequency. What is the difference between post-sepsis syndrome and recovery - could these categories be collapsed? Include # (%) over the bars.

Response:

Thank you for this suggestion. We have re-ordered by frequency and included the percentage above the bars in the updated Figure 3. We believe that post-sepsis syndrome and recovery are distinct and best considered separately. Recovery refers to the overall healing process, while post-sepsis syndrome describes ongoing physical and mental health challenges that some survivors experience.

Figure 5: Needs a footnote with acronym meanings (EDID?) and also what "Other" includes. Include # (%) over the bars.

Response:

We have added footnotes to clarify EDID and what Other entails, and have included the percentage over the bars in the updated Figure 4.

DISCUSSION:

Line 275: I am unsure what this sentence means?

Response:
We have rewritten the sentence as follows: *"Despite an increase in the number of researchers interested in sepsis research, there was a lack of consolidated understanding of the trends in sepsis research, funding, and diversity of researchers."* We hope this is clearer.

Line 284: I would also mention that nobody seems to be working on this in the territories.
I think it is worth discussion that most respondents are senior faculty - why might this be? Should we worry about our pipeline of junior sepsis researchers?

Response:
Thank you for the suggestions. We have mentioned in the Discussion that no one is currently working on sepsis in the territories. Additionally, we have discussed the gap in junior sepsis researchers entering the pipeline, highlighting the concern. We also emphasize that Sepsis Canada is actively addressing this gap by investing in the expansion of the LifTING Training Program to support the development of sepsis research trainees. (*I am not sure if adding this seems a bit grandiose)*

Consider discussing strategies to improve DEI in sepsis research.

Response:
Thank you for this thoughtful suggestion. While discussing strategies to improve DEI in sepsis research is an excellent idea, it goes beyond the scope of the current manuscript. However, we recognize the importance of this issue and hope it will be addressed in future research on sepsis.
